# A Novel Early Diagnosis System for Mild Cognitive Impairment Based on Local Region Analysis: A Pilot Study

**DOI:** 10.3389/fnhum.2017.00643

**Published:** 2018-01-09

**Authors:** Fatma E. A. El-Gamal, Mohammed M. Elmogy, Mohammed Ghazal, Ahmed Atwan, Manuel F. Casanova, Gregory N. Barnes, Robert Keynton, Ayman S. El-Baz, Ashraf Khalil

**Affiliations:** ^1^Faculty of Computers and Information, Information Technology Department, Mansoura University, Mansoura, Egypt; ^2^BioImaging Laboratory, Department of Bioengineering, University of Louisville, Louisville, KY, United States; ^3^Department of Electrical and Computer Engineering, College of Engineering, Abu Dhabi University, Abu Dhabi, United Arab Emirates; ^4^School of Medicine, University of South Carolina, Greenville, SC, United States; ^5^University of Louisville Autism Center, Department of Neurology, University of Louisville, Louisville, KY, United States; ^6^Department of Bioengineering, University of Louisville, Louisville, KY, United States; ^7^Department of Computer Science and Information Technology, College of Engineering, Abu Dhabi University, Abu Dhabi, United Arab Emirates

**Keywords:** AD, CAD, PiB-PET, statistical analysis, personalized diagnosis

## Abstract

Alzheimer's disease (AD) is an irreversible neurodegenerative disorder that accounts for 60–70% of cases of dementia in the elderly. An early diagnosis of AD is usually hampered for many reasons including the variable clinical and pathological features exhibited among affected individuals. This paper presents a computer-aided diagnosis (CAD) system with the primary goal of improving the accuracy, specificity, and sensitivity of diagnosis. In this system, PiB-PET scans, which were obtained from the ADNI database, underwent five essential stages. First, the scans were standardized and de-noised. Second, an Automated Anatomical Labeling (AAL) atlas was utilized to partition the brain into 116 regions or labels that served for local (region-based) diagnosis. Third, scale-invariant Laplacian of Gaussian (LoG) was used, per brain label, to detect the discriminant features. Fourth, the regions' features were analyzed using a general linear model in the form of a two-sample *t*-test. Fifth, the support vector machines (SVM) and their probabilistic variant (pSVM) were constructed to provide local, followed by global diagnosis. The system was evaluated on scans of normal control (NC) vs. mild cognitive impairment (MCI) (19 NC and 65 MCI scans). The proposed system showed superior accuracy, specificity, and sensitivity as compared to other related work.

## 1. Introduction

Alzheimer's disease (AD) is a chronic neurodegenerative disorder marked by cognitive and behavioral impairments (Hodler et al., 2012; WHO, 2017). Statistically, 42% of AD sufferers are people over 85 years of age with the percentage decreasing to only 6% for people of 70–74 years old. Although the probability is small, younger individuals may also be affected (Brown, 2013).

AD is characterized by clinical symptoms and pathological features, both of which vary among patients (Lu and Bludau, 2011). In the clinical presentation, the patient faces progressive deficits in cognition as well as disturbances in thought, perception, and behavior. Neuropathological abnormalities include the formation of neurofibrillary tangles and neuritic plaques as well as neuronal loss and granulovacuolar degeneration. Indeed, the quantity and location of neurofibrillary tangles and neuritic plaques represent neurodegenerative features that distinguish AD from other types of dementia. Efforts to establish an early diagnosis of AD have been thwarted by the fact that pathological features of the disease occur 10–15 years before the emergence of clinical symptoms.

Mild cognitive impairment (MCI) can lead to AD. MCI can be defined as an impairment of cognition that is more severe than expected from normal aging and a persons education with objective evidence of impairment in one or more cognitive domains including memory, executive function, attention, language, or visuospatial skills. MCI does not interfere with his/her independence and daily activities including social or occupational functioning (Yaffe, 2013). In this regard, MCI represents an intermediate stage between the cognitive decline observed with normal aging and the severe impairment observed in dementia (Anderson et al., 2012; Yaffe, 2013). It is important to note that not all MCI cases proceed to AD, although studies have found that it increases the risk of later developing AD (Anderson et al., 2012; NIH, 2017). MCI due to AD is a progressive decline in cognition over months to years. MCI due to AD has a lack of significant vascular factors, vascular imaging findings, parkinsonism, visual hallucinations, prominent behavioral, or language disorders (Langa and Levine, 2014).

There are a number of tests that have to be considered when trying to establish a diagnosis of AD. These tests include: neuropsychological screening (to measure related cognitive impairments), patients medical history, and mental/physical examination. In addition, blood tests as well as brain imaging are usually evaluated to rule out other neurological or physiological disorders (Turkington and Harris, 2001; Wegrzyn and Rudolph, 2012). In general, these tests help to classify the subjects along the disease cascade into MCI, when the appearance of some cognitive decline does not fulfill dementia criteria, and other stages of AD (Wegrzyn and Rudolph, 2012). A recent meta-analysis of neuropsychological measures suggested that verbal memory measures and other language tests yield high predictive accuracy for those MCI subjects who will progress to AD. Other domains including executive function and visual memory showed better specificity than sensitivity (Belleville et al., 2017). These data show that there is a clinical need to identify biomarkers of neural circuits involved in MCI, which ultimately lead to the development of AD.

Brain biomarkers have been postulated to help in the diagnosis of AD throughout the diseases natural history. For example, Jack et al. (2010) presented a study in which positron emission tomography (PET) amyloid imaging and cerebrospinal fluid (CSF) amyloid beta 42 (Aβ42) revealed Aβ abnormalities in the brain. These abnormalities are the earliest pathological features observed in the AD-related disease cascade. Additionally, the study reported that both the increase in CSF tau and cerebral atrophy serve as biomarkers of neuronal injury and neurodegeneration. Finally, the decrease in 2-[^18^F]fluoro-2-deoxy-D-glucose PET (FDG-PET), as demonstrated by the study, helps in revealing the synaptic dysfunction that accompanies the neurodegeneration. Therefore, according to this study, sMRI measures abnormalities of the brain's structure, and FDG-PET/CSF-tau identifies tau-mediated neuronal injury and dysfunction. The authors concluded that PET amyloid imaging could be considered an early identifier of AD-related abnormalities.

PET is a main scanning application of the emission computed tomography (ECT) methodology. Despite the role of the PET amyloid imaging in the early diagnosis of AD, arguments for and against the implementation of this scan modality in clinics should be carefully considered. False positive diagnoses of AD may occur since normal elderly subjects can have elevated Aβ levels. Fortunately, the introduction of carbon-11 labeled Pittsburgh compound B (^11^C PiB), a neutral analog of the thioflavin T, caused a noteworthy conversion in studies related to AD (Johnson et al., 2013). The compound assists in visualizing the pathological hallmarks related to AD and consequently helps in quantitating the neuropathological burden during subsequent AD stages (Varghese et al., 2013).

Numerous research efforts have been proposed to help in the differentiation between normal control (NC), MCI, and AD utilizing PET scans. These efforts include studies focused on testing computer-aided diagnosis (CAD) systems, such as the automatic classification system proposed by Illán et al. (2010). For this purpose, principal component analysis (PCA) and support vector machines (SVM) were utilized. To evaluate the system, the PiB and FDG related scans were used to compare their results regarding early diagnosis. Although PiB and FDG showed similar accuracies, the PiB had a higher power of discrimination in the very early cases. Again, Illán et al. (2011) utilized PET scans to construct a CAD system that relied on eigenimage framework and composed of feature extraction, dimensionality reduction, and classification stages. In this system, PCA and independent component analysis (ICA) were utilized for image projection (feature reduction), eigenimage based decomposition for feature reduction, and SVM for classification. Through these stages, the system achieved an accuracy of 88.24%. Another study by Jiang et al. (2015) demonstrated a CAD system that improved the classification accuracy of AD using the PCA, ICA, and SVM. The PCA was used for dimension reduction, ICA for feature extraction, and SVM using linear and radial basis function (RBF) kernels for classification. The results of their study showed either better or equivalent performance as compared to the competitive CAD methods with higher accuracy than the traditional visual assessment methods.

In the same context, López et al. (2011) proposed a CAD system aimed at improving the accuracy of three-way classification between NC, MCI, and AD. The study used PCA and linear discriminant analysis/Fisher discriminant ratio for the feature selection process followed by artificial neural network/SVM for the classification purpose. Testing this methodology with FDG PET scans led to a classification accuracy of 89.52%. Martínez-Murcia et al. (2012) presented a CAD system composed of three stages: Mann-Whitney-Wilcoxon *U*-Test for voxel selection in order to exclude outliers, factor analysis for feature extraction, and linear SVM for classification. Testing this system on PET scans achieved an accuracy of 92.9%. Also, Chaves et al. (2012) exploited association rule mining in a CAD system used in the early diagnosis of AD. Testing the system using two datasets, including PET scans, showed better results as compared to other related work. Padilla et al. (2012) introduced a CAD system to serve the early diagnosis of AD by combining nonnegative matrix factorization (NMF) for feature selection and reduction, and SVM with confidence bounds for classification. Application of the system led to an accuracy of 86%.

In addition to the aforementioned studies, various researchers have used voxel analysis for the early diagnosis of AD. For example, Morbelli et al. (2012) performed voxel-wise interregional correlations through statistical parametric mapping to extract relevant information. The study's results illustrated the association between the pathophysiological process of AD and alterations of the functional brain networks. According to the study, the default mode network (DMN) and memory function-related networks are the main causes of such alterations. Kemppainen et al. (2006) compared AD vs. NC subjects to find the brain regions that showed significant increases in the uptake of ^11^C PiB by applying voxel-based analysis. A Statistical Parametric Mapping (SPM) analysis was performed using automated region of interest (ROI). The study found that the voxel-based analysis showed widespread distribution regarding the increased ^11^C PiB uptake. Ziolko et al. (2006) statistically evaluated the amyloid imaging agents' (i.e., PiB) retention differences throughout the brain. In addition, they compared the PiB results with the FDG-based scans of glucose metabolism. The results revealed that the statistical significance of the PiB analysis was both greater than others and had a larger spatial extent. The results also showed that the PiB significance was retained after corrections of family-wise error and false discovery rate.

Forsberg et al. (2008) studied the amyloid deposition in patients with MCI. They used ^11^C PiB and FDG based PET scans with AD, which were compared with NC scans. The analysis showed an intermediate retention of the mean cortical PiB in the MCI as compared to NC and AD. Also, the study found significantly higher PiB retention in the MCI conversion to AD group, comparable to that of AD patients (*p* > 0.01) and much less in MCI subjects who did not convert to AD. Shin et al. (2010) presented a voxel-based analysis relying on the FDG, PiB along with another tracer known as 2-(1-6-[(2-(^18^F)fluoroethyl) (methyl) amino]-2-naphthylethylidene) malononitrile (FDDNP). These tracers were utilized to address the pathological hallmarks of AD, beta amyloid plaques, potential neurofibrillary tangles, and glucose metabolism related impairments. The experimental results demonstrated the available capacity to develop and test disease-modifying drugs targeting both tau and amyloid pathology, and/or energy metabolism when using the same subject based PET imaging with these three tracers.

Despite the achievements of the aforementioned investigations, the studies only supported a global diagnosis that indicate whether or not the subject belongs to a certain studied group. The main objectives of this paper are summarized in the following points. First, it provides a personalized diagnosis to help individualize diagnostic options as well as monitoring the disease progression. Second, it improves the final global diagnosis results as compared with previously published studies. To achieve these goals, our system utilized PiB PET scans due to the superior role of this brain imaging modality, as compared to other scanning modalities, when applied to the early diagnosis of the disease. Therefore, the paper is organized as follows. Section 2 (vide infra) starts by describing the materials used for the preparation of the paper and defines the methods used in the proposed CAD system. In section 3, different experimental results are presented to evaluate the performance as well as the efficiency of our system. Finally, the discussion of the applied tests and the future work are highlighted in section 4.

## 2. Materials and methods

### 2.1. Materials

A set of ^11^C PiB-PET scans were used to validate the proposed framework. These scans were collected from the Alzheimer's Disease Neuroimaging Initiative (ADNI) database (adni.loni.usc.edu). The database of ADNI was initially launched in 2003 as a public-private partnership, led by principal investigator Michael Weiner, MD. The goal of demonstrating ADNI was to test whether serial MRI, PET, or other markers, in addition to clinical and neuropsychological assessment, can measure the progression of MCI and AD by combining them. For further advance information, please see www.adni-info.org. The used dataset was obtained from ADNI 1 where it contains a total number of 84 scans obtained from 19 NC and 65 MCI subjects. NC comprises those subjects who do not show any signs of depression, cognitive impairment, or dementia. The MCI group, in general, comprises those subjects with subjective memory concerns, whether self-reported or through an informant or a clinician. Those subjects display neither significant impairment levels in other cognitive domains nor signs of dementia. The Logical Memory II subtest of the Wechsler Memory Scale (WMS) was performed on the participants to document the normality/abnormality of their memory function with respect to their level of education. The demography of the used dataset is presented in Table 1.

**Table 1:** The demographic data of the NC and MCI groups of the ^11^C PiB-PET scans.

**(*N* = 84)**	**Average Age ± SD**	**Gender N (%)**		**WMS logical memory II results (based on years of education)**		
		**Male**	**Female**	**≥16 years**	**8–15 years**	**0–7 years**
NC (19)	78.3 ± 5.01	11 (57.89)	8 (42.1)	≥ 9	≥ 5	≥ 3
MCI (65)	75.78 ± 7.67	44 (67.69)	21 (32.30)	≥ 8	≥ 4	≥ 2

### 2.2. Methods

**Figure 1:**
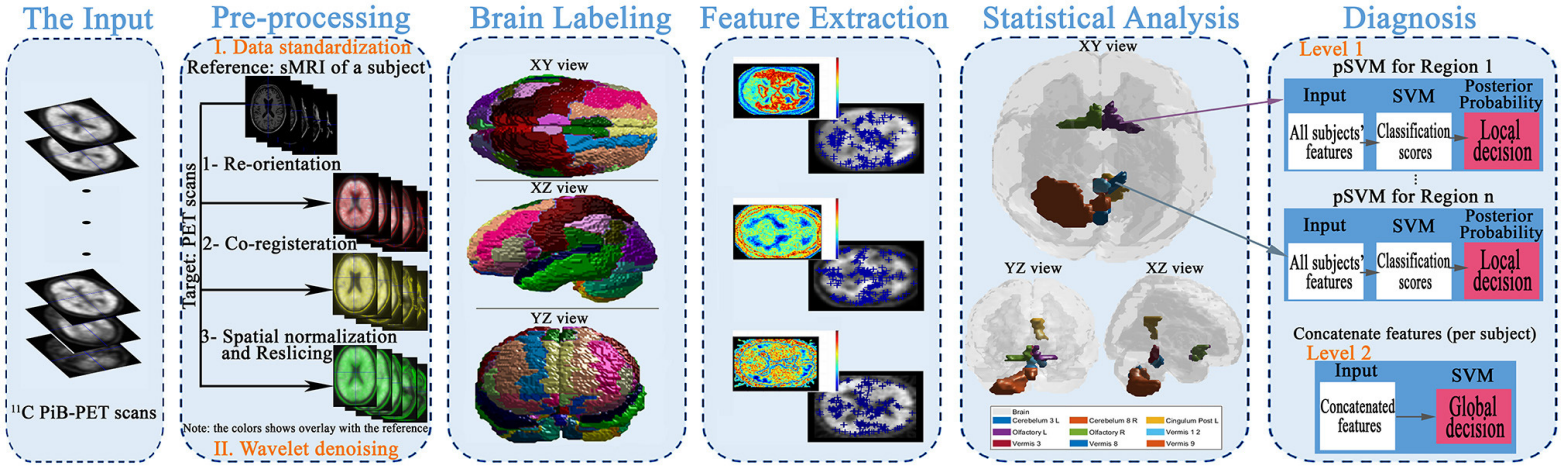
The framework of the proposed early diagnosis system for AD based on local and global region analysis using ^11^C PiB PET scans.

The main aim of this article is to present local (i.e., region) based diagnosis of the MCI regarding AD, to assist clinicians in the personalized treatment of the disease. To achieve this goal, five main steps were performed, as illustrated in Figure 1. First, the scans were preprocessed through data standardization and de-noising. Data standardization aimed to prepare the scans for the labeling step, while the de-noising process aimed to improve the scan's quality and consequently the systems accuracy. Second, the atlas of Automated Anatomical Labeling (AAL) was used for brain parcellation to serve the local diagnosis goal. After labeling the brain, we used a Laplacian of Gaussian (LoG) with the automatic scale to extract the discriminant features from the scans. Then, a statistical analysis was performed to determine the significant brain regions to analyze rather than using all the labeled regions in the decision-making process. Finally, these regions were used to construct two decision-making levels using a probabilistic version of SVM (pSVM) and standard SVM to provide local followed by global diagnosis. The details of the proposed system are presented in the following subsections.

#### 2.2.1. Preprocessing

Scans underwent some preprocessing to orient the data and reduce noise. For orienting the data in a standard coordinate system, the SPM MATLAB toolbox (NeuroImaging, 2017) was used to perform re-orientation, co-registration, spatial normalization, and re-slicing. Noise reduction was accomplished through wavelet shrinkage. Details are as follows: PET scans' associated sMRI data were re-oriented so that the anterior-posterior axis coincides with the AC-PC line. This differs from the ADNI pipeline, which only ensures the axis is parallel to the AC-PC line. The associated PiB-PET scan was re-oriented to the resulting sMRI scan to produce a re-orientation matrix that was then used to re-orient the remaining PiB-PET scans. Precise co-registration between the PET scans and the previously used sMRI scan was performed using rigid body transformations (translations and rotations) to maximize the mutual information. Then, the spatial normalization and re-slicing were applied to the sMRI and PET scans to align the scans to the MNI-152 standard space. In this step, general affine transformation (translations, rotations, non-uniform scaling, and shears) was used, followed by nonlinear deformations. After data standardization, wavelet based de-noising was applied using the symlet8 mother wavelet with Steins unbiased risk estimate as a threshold selection rule and soft thresholding (Bagci and Mollura, 2013). The aim was to retain image detail while removing artifacts of image acquisition and/or transmission (Agrawal and Bahendwar, 2011). At this point, the scans were ready for voxel-wise comparison and labeling steps.

#### 2.2.2. Brain labeling

Due to the local diagnosis based goal of the proposed system, the brain labeling/partitioning needs to be performed. Through this aim, a detailed diagnosis of the subjects could be achieved. For this purpose, any of the detailed based brain atlases can be used, such as AAL, Talairach Daemon, and Brodmann areas atlases (Su et al., 2014; Zhang et al., 2015; Salas-Gonzalez et al., 2016). In this paper, the AAL atlas was used to label each of the preprocessed scans voxel's positions to the matched anatomical regions. The AAL atlas provides a total of 116 brain regions: 45 per cerebral hemisphere, 9 per cerebellar hemisphere, and 8 in the vermis of the cerebellum. AAL provides a detailed parcellation of the brain and is recommended for use with PET scans. To accomplish the labeling procedure, the xjView MATLAB toolbox (Alivelearn.net, 2017) was utilized.

#### 2.2.3. Blob detection based feature extraction

**Figure 2:**
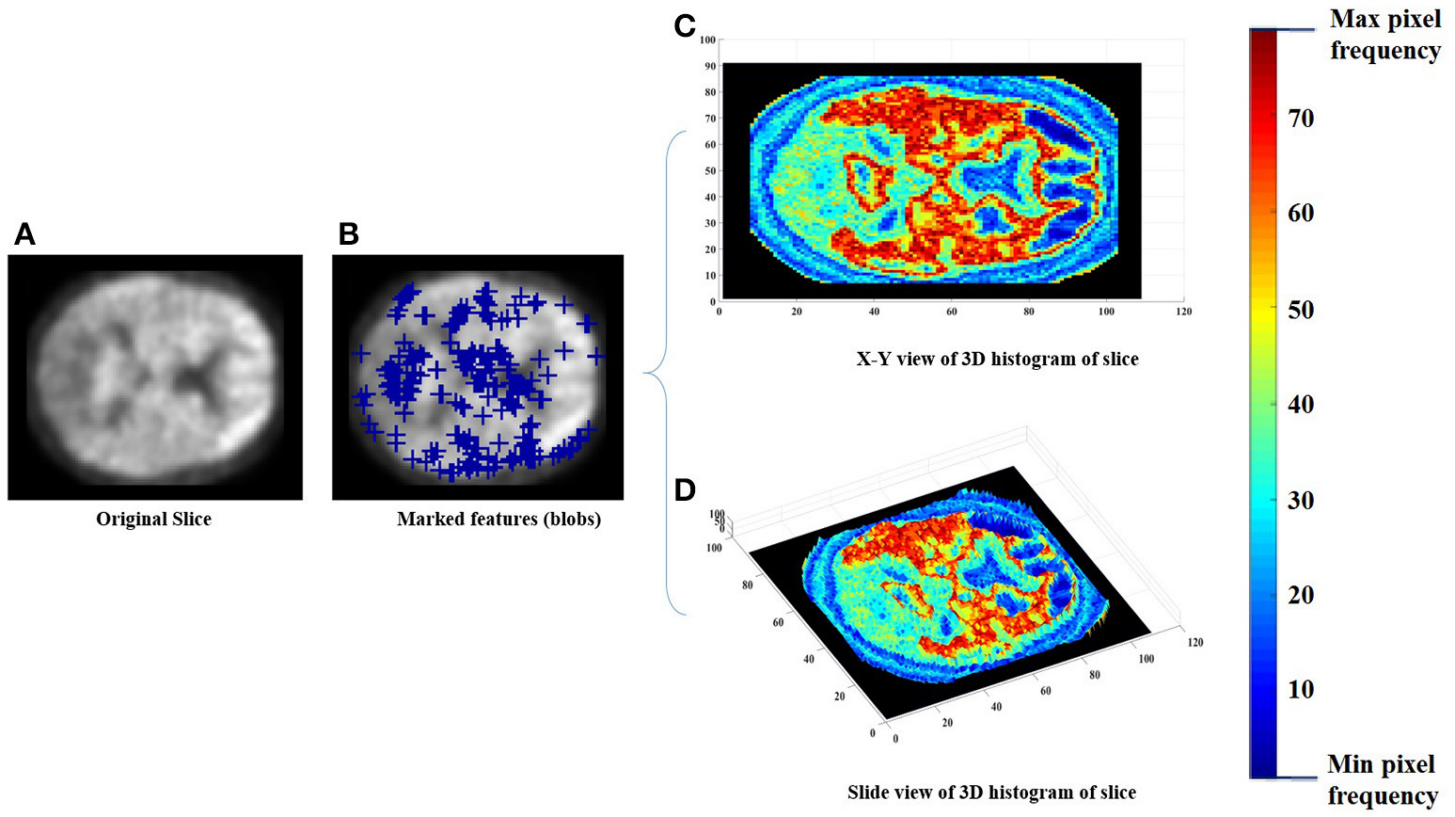
An example of the feature extraction step, **(A)** the original slice, **(B)** the obtained features (blue pluses), **(C,D)** a 3D histogram representation of the slice to indicate the targeted local maxima/minima locations.

Each of the labeled regions is individually fed to the scale-invariant blob detector, which employs LoG with automatic scale selection, for the purpose of feature extraction. Blob detection aims to separate structures (i.e., blobs) from the image background. Each blob is itself a radially symmetric distribution of image intensity about a local minimum or maximum (Toennies, 2012). Blobs corresponding to local maxima could reveal the targeted abnormalities, given that significantly greater retention of PiB in a brain region is linked with greater incidence of Aβ plaques within that region (Shin et al., 2010). Figure 2 shows a sample of the extracted features with a 3 Dimensional (3D) smoothed histogram showing the local maxima locations that are targeted through the detector.

#### 2.2.4. Statistical analysis

AAL regions where mean PiB uptake differs significantly in MCI with respect to NC was determined using two-sample *t*-tests. Each atlas region was tested independently. The Bonferroni method was applied to identify a region as “significant” when the *p*-value was less than 0.00043 (i.e., 0.05/116). Significant regions were subsequently used in building a classifier.

#### 2.2.5. Diagnosis

A two-level diagnosis was performed to make local (region-specific abnormalities) and global (level of cognitive impairment) diagnoses. For this purpose, SVM and one of its variant (pSVM) were utilized. Standard SVM is an abstract machine learning technique where the training data are used for the learning followed by a generalization attempt for correct prediction on other novel data (Campbell and Ying, 2011). In SVM, a hyperplane (known as maximal margin hyperplane) is used for the binary separation of the labeled training data. The goal is to build a decision function *f*:*R*^*S*^ → ±1, according to S-dimensional training patterns *p*_*i*_ and *t*_*i*_, capable to perform classification for new example (p, t): $\left(p_{1},t_{1}\right),\left(p_{2},t_{2}\right),\ldots ,\left(p_{s},t_{s}\right)\in R^{S}\pm 1$. The decision hyperplanes in multidimensional feature space can be defined through either using a linear separation of the training data, using linear discriminant functions, or combining SVM with kernel techniques that produce a non-linear decision boundary (hyperplane) in the input space (Illán et al., 2011). Beside its classification power, a variation of SVM, which produces a posterior probability output of the classifier (pSVM), is useful to allow further post processes.

In the proposed system, a separate pSVM model was constructed, in the first diagnosis level, for each significant region. Each pSVM produces a probabilistic result for the incidence of MCI given the features from its brain region independently of all others. In the second level, the scores obtained from the first level were fused with respect to each subject and used to train and test a single SVM model to produce the global diagnosis. Classifier performance, i.e., accuracy, sensitivity, and specificity, were estimated using both leave-one-subject-out (LOSO) and K-fold cross-validation, with two- and four-fold.

## 3. Results

**Figure 3:**
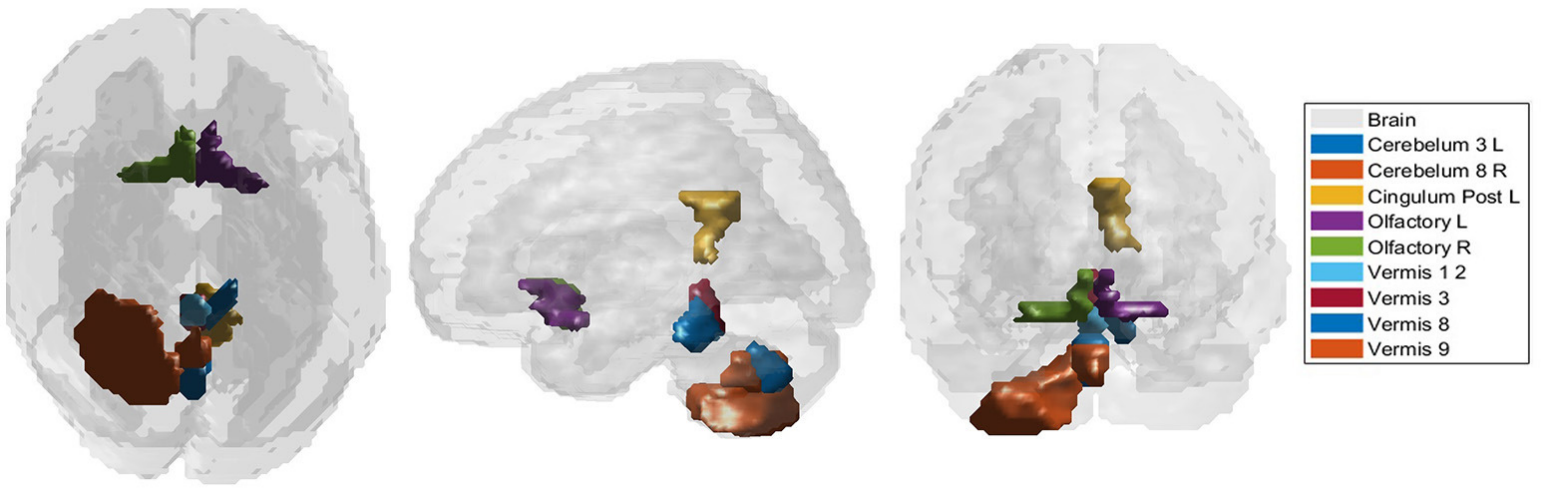
The significant regions as obtained through the two sample *t*-test.

According to the Bonferroni corrected two-sample *t*-tests, the AAL brain regions of significance, nine regions, were Cerebelum_3_L (left alar central lobule), Cerebelum_8_R (right biventer lobule), Cingulum_Post_L (left posterior cingulate gyrus), Olfactory_L and Olfactory_R (bilateral olfactory cortex), plus Vermis_1_2, Vermis_3, Vermis_8, and Vermis_9 (lobules I, II, III, VIII, and IX of the vermis) as visualized in Figure 3. The performance of the resulting classifier was evaluated under a number of different kernels selected for the SVM, with best results being obtained when the linear kernel was used at both level 1 and level 2 (Table 2). This classifier was used to compare three cases, using data from all brain regions, using all regions except the significant ones, and using pre-selected, specific regions. The third case found to outperform the other two cases (Table 3).

**Table 2:** Evaluation of SVM classifier performance when different kernels are used.

			**Level 2**								
			**LOSO**			**K-fold**					
						***K* = 2**			***K* = 4**		
			**RBF**	**Linear**	**Polynomial**	**RBF**	**Linear**	**Polynomial**	**RBF**	**Linear**	**Polynomial**
**Level 1**
	**RBF**	ACC	72.61	50	22.61	59.52	22.61	77.38	77.38	22.61	77.38
		Spec.	26.31	57.89	100	57.89	100	0	0	100	0
		Sens.	86.15	47.69	0	60	0	100	100	0	100
	**Linear**	ACC	77.38	**88.09**	95.23	77.38	**79.76**	77.38	77.38	**89.28**	77.38
		Spec.	0	**47.36**	78.94	0	**10.52**	0	0	**52.63**	0
		Sens.	100	**100**	100	100	**100**	100	100	**100**	100
	**Polynomial**	ACC	77.38	88.09	77.38	77.38	80.95	76.19	77.38	88.09	77.38
		Spec.	0	47.36	0	0	26.31	0	0	47.36	0
		Sens.	100	100	100	100	96.92	98.46	100	100	100

*Classifier accuracy (ACC), sensitivity (Sens.) and specificity (Spec.), respectively in %, were estimated over all the labeled regions using LOSO and K-fold cross-validation. The bolded results (linear-pSVM linear-SVM) indicates the best combination of kernels used in the two levels*.

**Table 3:** Using LOSO and K-fold cross-validation methods to evaluate the classification results, in %, (using linear-pSVM linear-SVMs) using the features of different cases of input regions: all the labeled regions, all the regions except the significant ones, and the significant regions only.

		**LOSO**	**K-fold**	
			***K* = 2**	***K* = 4**
All labeled regions	ACC	88.09	79.76	89.28
	Spec.	47.36	10.52	52.63
	Sens.	100	100	100
The resulting significant regions	ACC	100	97.61	98.80
	Spec.	100	94.73	94.73
	Sens.	100	98.46	100
Excluding the significant regions	ACC	82.14	77.38	83.33
	Spec.	21.05	0	26.31
	Sens.	100	100	100

**Figure 4:**
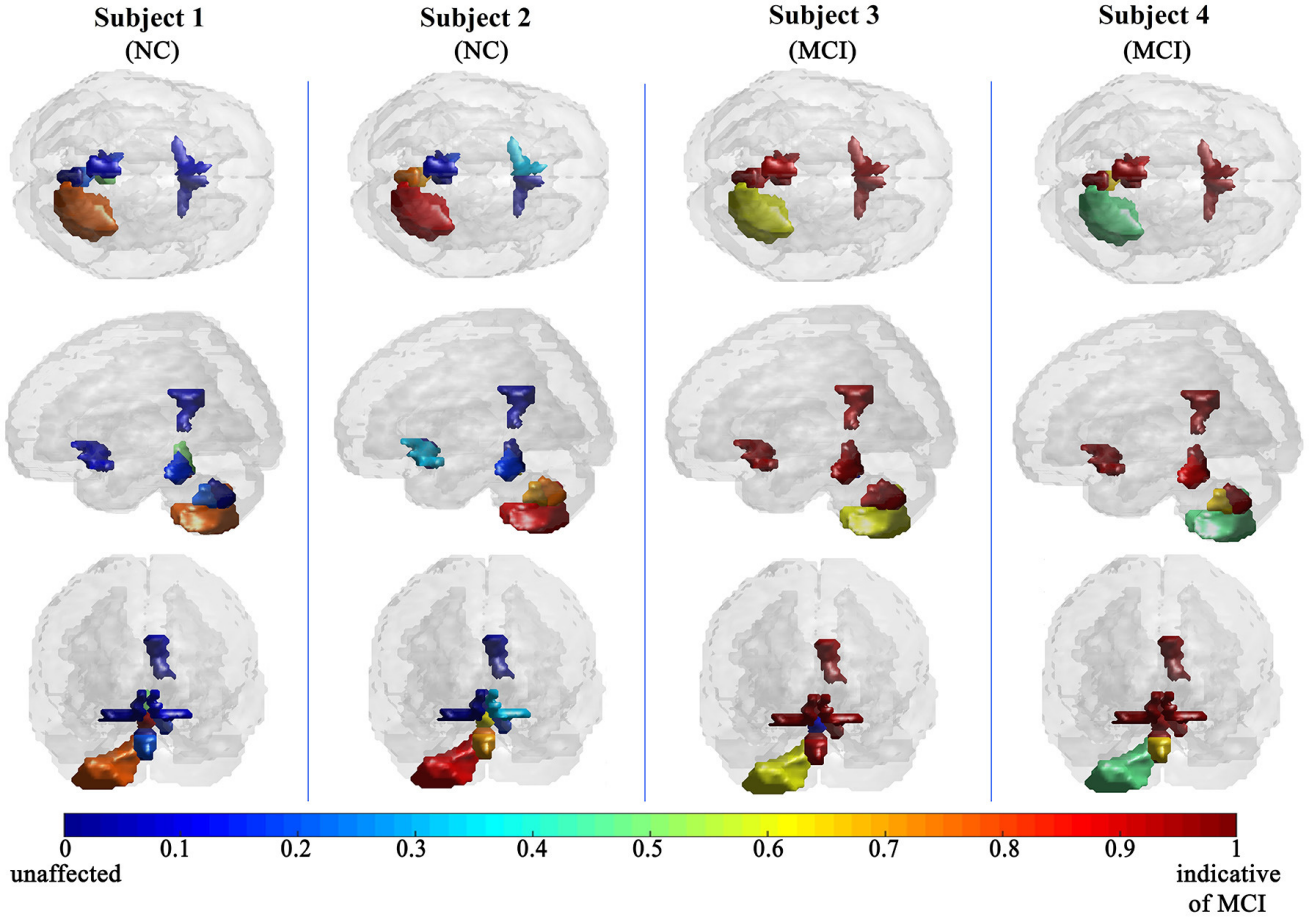
Examples of local diagnosis results for NC vs. MCI classification problem.

**Figure 5:**
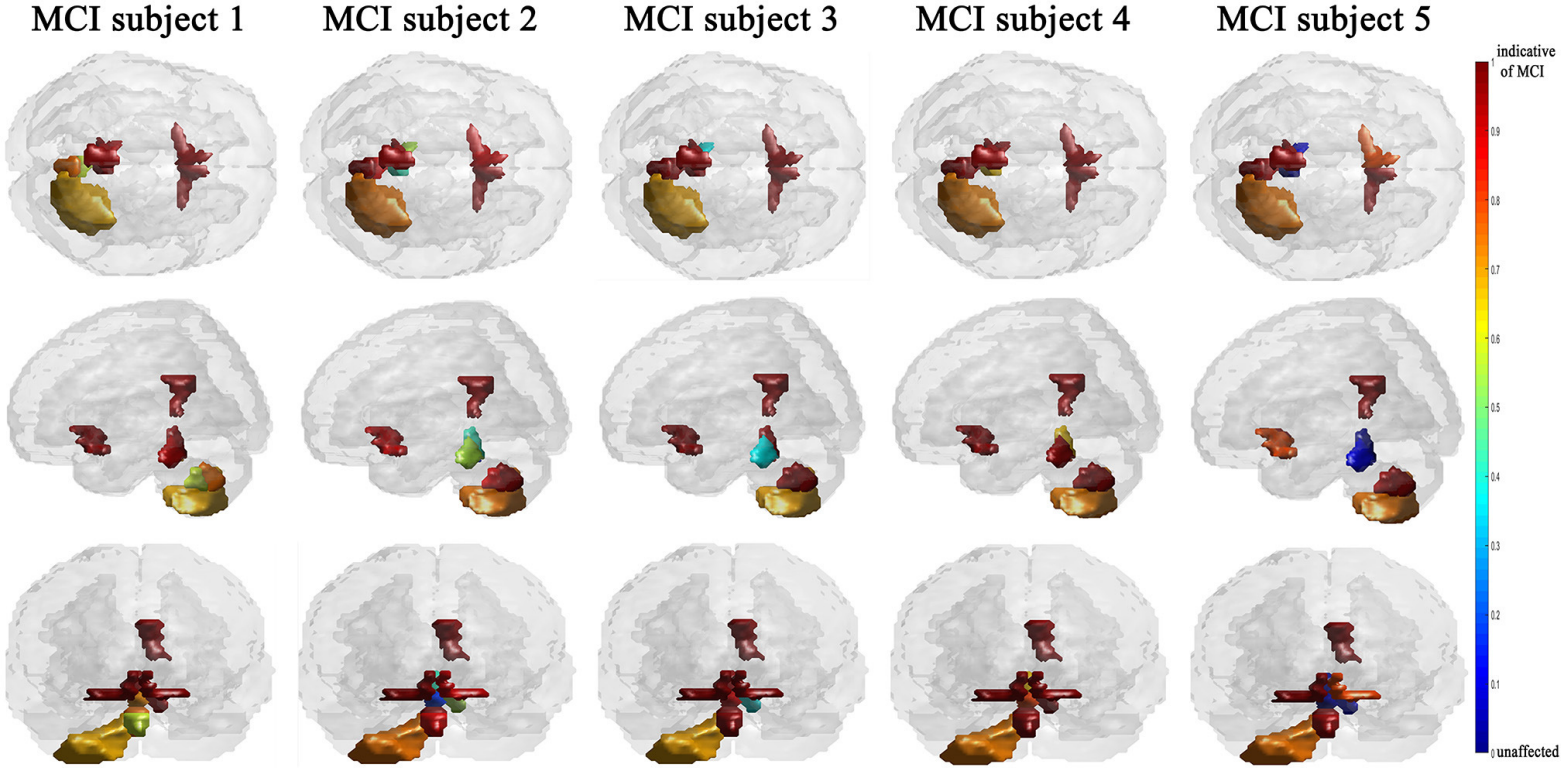
Examples of local diagnosis results of different MCI subjects.

The efficiency of the proposed system was compared to two other published methods (Chaves et al., 2012; Jiang et al., 2015). Our methodology was found to distinguish MCI from NC as well or better than either previous techniques (Table 4). Note that performance of the compared classifiers is taken directly from the respective publications since each of them used the same database (i.e., ^11^PiB PET scans from ADNI), and LOSO cross-validation. Finally, Figures 4, 5 provide examples of the local diagnostic results (i.e., the results of the first diagnosis level). Figure 4 illustrates the local diagnosis of two NC and two MCI cases. While Figure 5 shows examples of five different MCI subjects. The color bar in both figures represent the degree of abnormality starting from 0 (unaffected) to 1 (indicative of MCI). These examples indicate the varying abnormality effects in each significant region for each case independently. The implementation of the proposed CAD system can be found in the Supplementary Material.

**Table 4:** The comparison of the proposed system's performance results, in %, against other related studies using LOSO cross-validation method.

	**ACC**	**Spec**.	**Sens**.
Chaves et al., 2012	90.48	100	87.69
Jiang et al., 2015	89.17	–	–
The proposed system	100	100	100

## 4. Discussion

This paper discusses a personalized MCI diagnosis system while improving the diagnostic performance as compared to other available methods. The personalized diagnosis is achieved through regional/local measurements, using the AAL atlas, which reflects how the disease affects different brain regions. To enhance this procedure, a statistical analysis was initially performed to determine the salient brain regions and consequently analyze the influence of the disease on them through the first diagnosis level, as shown in Figures 4, 5. Apart from being the first stage of the MCI diagnosis procedure, the reported local diagnoses are helpful assistance in the personalized management of the disease. This has been represented through the color bar that shows the degree of abnormality from 0 (unaffected) to 1 (indicative of MCI) in each one of the nine significant regions separately. Finally, the system fuses the regional based probabilistic results obtained from the first diagnosis level to produce the final global diagnosis of each subject.

Regarding the cerebellar regions identified as significant, there have been several studies that focused on cerebellar abnormalities in dementia. Some of these studies were neuropathological-based studies that found that the neuronal shrinkage and loss represent well-known changes that accompany AD (Baldaçara et al., 2011). Morphological studies have targeted the prominent neuropathological AD-related hallmarks, like amyloid plaques (Cole et al., 1993; Wang et al., 2002). According to Wang et al. (2002), most AD-based pathological features that are found in the cerebellum include diffuse Aβ deposits. Considering the later finding and since the regions with increased Aβ plaques are represented as the high retention in the PiB, we could justify this finding in the early stage of AD. In addition to these findings and according to Sjöbeck and Englund (2001), structural based changes, mainly involved in the vermis, were judged to represent the progression of the disease.

Dysfunction in the olfactory cortex was found, through some studies, to probably be one of the earliest symptoms that are clinically obtained regarding AD (Serby et al., 1991; Devanand et al., 2000). According to Velayudhan (2015), the combination of the olfactory function tests along with the conventional diagnostic methods provides the ability to improve the sensitivity as well as the specificity of diagnosing AD. This consequently facilitates both the early recognition and diagnosis of AD. More details about the research progress and the future directions of the olfactory dysfunction in AD could be found in Zou et al. (2016).

Finally, as regards the posterior cingulate gyrus, some neuroimaging studies, which targeted several cortical regions, have identified that the posterior cingulate regions of the medial parietal cortex are among the earliest regions affected in AD (Scheff et al., 2015). These studies include one that supports the involvement of the posterior cingulate in the very early progression of AD (Rami et al., 2012).

In general, as shown in Table 2, the linear kernel shows best results either using it on both levels of the classifier or along with the polynomial kernel, while the RBF kernel performed poorly even when used conjointly with another kernel. The ability of the extracted features to differentiate the two groups (NC and MCI) and consequently make them linearly separate could justify the highest results of the linear kernel. For the polynomial as well as the RBF kernels, nonlinear kernels, the obtained separable features in addition to the small size of the dataset caused the outperformance of the polynomial kernel as compared to the RBF kernel that can show better results with large size of the dataset. According to these results and since linear-linear SVM-based classifiers show in general the best results, they were used to build the CAD system.

As expected, the significant region-based diagnostic performance outperforms that of the other two trial classifiers (Table 3) with a maximum performance of 100% of accuracy, specificity, and sensitivity. This finding could be due to the discrimination power of the features extractor in addition to the demonstration of alpha that helped in obtaining these salient regions. Regarding the two other tests, using all the labels shows better results than excluding the significant regions but worse than using the significant regions. This finding could be due to the presence of the labeled regions that are not significant enough to differentiate between the groups and consequently led to misclassification results that could finally affect the overall performance of the system. In addition, this is the same case when excluding the significant regions, but in addition to the presence of these not well significant regions, the significant regions are excluded causing this drop of performance results as compared with the other two tested cases.

The fact that our system improved performance with respect to other techniques while using the same dataset is of crucial consideration. The superior results of the proposed system over prior work (Table 4) could be justified through the capabilities of the combined components of the system that could extract the most discriminant features, identify the significant brains regions and then perform the classification using the SVM along with the linear kernel.

Since the primary goal of the paper was to provide local diagnosis, Figures 4, 5 illustrated various examples that demonstrate the variability of the abnormality in each of the salient regions among the subjects. Figure 4 showed sample cases of NC and MCI groups proving this point where the color bar indicates the degree of the diseases effect starting with the dark blue, no effect, to the dark red, total effect. Figure 5 shows additional illustration source of the local diagnosis. The figure includes five different MCI subjects where the abnormalities' degree differ from one subject to another. Both figures demonstrate the power of the proposed system to reveal the local diagnosis of the disease on the significant brain regions. The analysis results that could be derived from the figures show the ability of the proposed analysis to reflect the current degree of the dysfunctionality that each region achieved through the disease. This visualization consequently can be considered an effective assistant for the individualized/personalized diagnosis process.

According to the above results, the proposed system may be of assistance in the diagnosis of MCI. In other words, the system offers a personalized diagnosis of the subjects in a short computation time using a subset of the brain's regions rather than using all regions. Additionally, the system provides high global diagnosis results compared to other related studies. Due to the promising results of the proposed system and pilot nature of our data, we plan to examine two goals in the future work. First, the system's performance will be evaluated on larger PET scan datasets. Second, the results will be incorporated with other AD-related scanning modalities, to provide more analysis based assistance for the early diagnosis of AD.

## Author contributions

FE-G: writing the manuscript and conducting the experiments. ME: advising, designing the experiments, developing the algorithm, and reviewing the entire paper. MG, RK, and AK: advising and financial support. AA: advising and reviewing the entire paper. MC: emphasizing the clinical aspect of the paper and reviewing the entire paper. GB: advising, reviewing the clinical aspect of the paper, reshaping the paper, and extensively editing it. AE-B: advising, designed the experiments, developing the algorithm, and reviewing the entire paper.

### Conflict of interest statement

The authors declare that the research was conducted in the absence of any commercial or financial relationships that could be construed as a potential conflict of interest.
